# In Vitro Screening of East Asian Plant Extracts for Potential Use in Reducing Ruminal Methane Production

**DOI:** 10.3390/ani11041020

**Published:** 2021-04-04

**Authors:** Rajaraman Bharanidharan, Selvaraj Arokiyaraj, Myunggi Baik, Ridha Ibidhi, Shin Ja Lee, Yookyung Lee, In Sik Nam, Kyoung Hoon Kim

**Affiliations:** 1 Department of Agricultural Biotechnology, College of Agriculture and Life Sciences, Seoul National University, Seoul 08826, Korea; bharanidharan7@snu.ac.kr (R.B.); mgbaik@snu.ac.kr (M.B.); 2 Department of Food science and Biotechnology, Sejong University, Seoul 05006, Korea; arokiyaraj16@gmail.com; 3 Department of Ecofriendly Livestock Science, Institute of Green Bio Science and Technology, Seoul National University, Pyeongchang 25354, Gangwon-do, Korea; ridha@snu.ac.kr; 4 Institute of Agriculture and Life Science & University-Centered Labs, Gyeongsang National University, Jinju 52828, Gyeongsangnam-do, Korea; tlswk1000@hanmail.net; 5 National Institute of Animal Sciences, RDA, Jeonju 54875, Jeollabuk-do, Korea; yoo3930@korea.kr; 6 Research Centre for Environment Friendly and Quality Livestock Production and Technology, Hankyoung National University, Anseong 17579, Gyeonggi-do, Korea; isnam@hknu.ac.kr; 7 Department of International Agricultural Technology, Graduate School of International Agricultural Technology, Seoul National University, Pyeongchang 25354, Gangwon-do, Korea

**Keywords:** in vitro, screening, methane, tannin, saponin, unsaturated fatty acids, protozoa

## Abstract

**Simple Summary:**

Methane from ruminants is a major contributor to total greenhouse gases. Therefore, ruminant nutritionists have proposed strategies to mitigate methane emissions, such as chemical inhibitors and ionophores. However, dietary manipulation including natural feed additives is more practical, considering consumer preferences. Therefore, the current experiment screened 137 plant species, indigenous to East Asian countries, to select novel anti-methanogenic candidates as natural feed additives. Among these species, an extract from the seeds of *Pharbitis nil* exhibited a maximum 37% reduction of methane in a conformation assay. Identification of active compounds present in the seeds of *Pharbitis nil* revealed enrichment of polyunsaturated fatty acids, which were dominated by linoleic acid (18:2). The extract had negative effects on the populations of ciliated protozoa and H_2_-producing *Ruminococcus flavefaciens*, thereby increasing the proportion of propionate, similar to the effect of monensin. This is the first report to suggest that the seeds of *P. nil* could be a promising anti-methanogenic alternative to ionophores or oil seeds.

**Abstract:**

Indiscriminate use of antibiotics can result in antibiotic residues in animal products; thus, plant compounds may be better alternative sources for mitigating methane (CH_4_) production. An in vitro screening experiment was conducted to evaluate the potential application of 152 dry methanolic or ethanolic extracts from 137 plant species distributed in East Asian countries as anti-methanogenic additives in ruminant feed. The experimental material consisted of 200 mg total mixed ration, 20 mg plant extract, and 30 mL diluted ruminal fluid-buffer mixture in 60 mL serum bottles that were sealed with rubber stoppers and incubated at 39 °C for 24 h. Among the tested extracts, eight extracts decreased CH_4_ production by >20%, compared to the corresponding controls: stems of *Vitex negundo* var. *incisa,* stems of *Amelanchier asiatica*, fruit of *Reynoutria sachalinensis*, seeds of *Tribulus terrestris*, seeds of *Pharbitis nil*, leaves of *Alnus japonica*, stem and bark of *Carpinus tschonoskii*, and stems of *Acer truncatum*. A confirmation assay of the eight plant extracts at a dosage of 10 mg with four replications repeated on 3 different days revealed that the extracts decreased CH_4_ concentration in the total gas (7–15%) and total CH_4_ production (17–37%), compared to the control. This is the first report to identify the anti-methanogenic activities of eight potential plant extracts. All extracts decreased ammonia (NH_3_-N) concentrations. Negative effects on total gas and volatile fatty acid (VFA) production were also noted for all extracts that were rich in hydrolysable tannins and total saponins or fatty acids. The underlying modes of action differed among plants: extracts from *P. nil, V. negundo* var. *incisa, A. asiatica*, and *R. sachalinensis* resulted in a decrease in total methanogen or the protozoan population (*p* < 0.05) but extracts from other plants did not. Furthermore, extracts from *P. nil* decreased the population of total protozoa and increased the proportion of propionate among VFAs (*p* < 0.05). Identifying bioactive compounds in seeds of *P. nil* by gas chromatography-mass spectrometry analysis revealed enrichment of linoleic acid (18:2). Overall, seeds of *P. nil* could be a possible alternative to ionophores or oil seeds to mitigate ruminal CH_4_ production.

## 1. Introduction

Ruminal methane (CH_4_) production is regarded as the cause of a loss of 3–10% of the gross energy intake of the animal and leads to the unproductive use of dietary energy [1]. Concerns regarding feed energy loss and climate change have led to many scientific studies aimed at lowering enteric CH_4_ production by ruminants through various mitigation options [2,3]. Notably, feed additives (e.g., CH_4_ analogues, hydroxymethylglutaryl-CoA reductase inhibitors, and nitrate and organic nitro compounds that are capable of decreasing rumen methanogenesis) have been extensively studied over the past two decades (reviewed in [4,5,6,7,8]). However, the use of certain chemically modified/synthesised compounds has adverse effects on fermentation at effective concentrations [4,5,6,7]. Intriguingly, 3-nitrooxypropanol is widely regarded as a promising candidate for enteric CH_4_ mitigation [9,10]. In addition to its potential to mitigate CH_4_, consumer preference may factor into the acceptance of such a synthetic compound if commercially available. Furthermore, concerns have been raised regarding the potential use of antibiotics because of their residues in final products, which have led to bans in the Republic of Korea since 2011 [11,12]. Therefore, natural plant feed additives that might be environmentally friendly and have a high level of acceptance among consumers are desired to improve livestock productivity.

Several studies have suggested that adding plant essential oils or plant extracts rich in plant secondary metabolites (PSM; e.g., tannins, saponins, and flavonoids) to ruminant diets may have beneficial effects on ruminal fermentation and CH_4_ production (reviewed in [13,14,15]). A comprehensive review by Patra et al. [4] also elaborated the direct and indirect roles of such PSMs against the growth and activity of rumen methanogens and the protozoan population. Similarly, numerous studies have shown that increasing fatty acid concentrations in the diet decreases CH_4_ production to a greater extent, but often exerts detrimental effects on digestibility and fermentation of feeds, as well as animal performance [16,17,18,19]. Therefore, it would be desirable to discover plant-based fatty acid-rich feed additives that decrease CH_4_ production, with additional effects of improved digestibility and animal performance.

Screening natural sources at a large scale is an initial step in the discovery and development of new compounds and feed additives. Few studies have performed screening experiments; these include the European Union project “Rumen-up” that evaluated 450 plants and plant extracts [20], 58 plants, herbs, and spices in Spain [21], 156 plants from natural grasslands in France [22], and 93 plant extracts in India [23]. Nevertheless, there is a persistent need to identify potential anti-methanogenic plants for the development of new compounds as natural feed additives, because many in vivo studies have shown adaptation of the additives by rumen microbes [24,25]. Furthermore, no study has focused on large-scale screening of plant species that are widely distributed in East Asian countries for their CH_4_ reduction potential. Hence, our objectives in this study were to screen 152 plant extracts from 137 plant species of East Asian origin for their potential to reduce CH_4_ production, in vitro; to study the effect on volatile fatty acids (VFAs) production, to quantify the bioactive compounds of the selected candidates; and to uncover their actions on methanogens, protozoa, and several other rumen cellulolytic bacteria using real-time polymerase chain reaction (PCR) analysis.

## 2. Materials and Methods

### 2.1. Plant Material

The Plant Extract Bank at the Korea Research Institute of Bioscience and Biotechnology (Daejeon, Korea) has stocked extracts of 1714 species of native Korean plants, which comprise 41% of all Korean plant species (excluding garden plants and food crops). In total, 6016 extracts from plants that are distributed in Korea and other East Asian countries are available at the Plant Extract Bank as an easy source to discover beneficial phytochemicals. Initially, 152 plant methanolic or ethanolic extracts from 137 plant species that were indigenous to East Asian countries were obtained, and their scientific names and the plant parts used for solvent extraction are listed in Table 1.

### 2.2. In Vitro Rumen Fermentation Assay

Two cannulated Holstein steers (mean body weight 680 ± 30 kg), cared for in accordance with the guidelines of the Animal Ethical Committee, Seoul National University, Republic of Korea (approval number SNU-160105-1), were used as rumen fluid donors. The animals were fed twice daily with 3.5 kg rice straw containing (k^−1^ dry matter [DM]) 857 g organic matter; 48 g crude protein; 26 g ether extract; 768 g neutral detergent fibre; 417 g acid detergent fibre; and 2.0 kg of commercial concentrate with (k^−1^ DM) 896 g organic matter, 156 g crude protein, 53 g ether extract, 310 g neutral detergent fibre, and 122 g acid detergent fibre. Ruminal digesta of approximately 800 mL was collected from each steer before the morning feeding and strained through four layers of muslin into a pre-warmed flask flushed with O_2_-free CO_2_. The fluid was diluted with O_2_-free buffer (adjusted to pH 7.0) [26] at a ratio of 1:2 (*v/v*) and placed in a water bath pre-heated to 39 °C with continuous CO_2_ flushing. Briefly, an in vitro screening assay was performed by incubating 20 mg of the extracts (dissolved in 1 mL of 10% dimethyl sulphoxide) with 30 mL of mixed rumen microorganisms in 60-mL serum bottles containing 200 mg DM of total mixed ration as the substrate. The ingredient and nutrient compositions of the substrate are provided in Table 2. The in vitro screening trial of all 152 plant extracts were tested in 2 different cycles with approximately 9-10 extracts per fermentation run with a total of 8 runs per cycle. Each run contained a control (i.e., with substrate and without plant extract), treatment (i.e., with substrate and 20 mg of plant extract), positive control (i.e., with substrate and 30 ppm of monensin; CAS No. 22373-78-0, Sigma-Aldrich, St. Louis, MO, USA), and three replicates. The bottles were sealed with rubber stoppers, covered with aluminium, and incubated at 39 °C for 24 h. After the completion of eight fermentation run (one cycle), potential candidates were chosen based on their abilities to decrease CH_4_ production by more than 20%, compared to their respective controls [21]. The same experimental procedure was followed for the screening assay in the second cycle. In vitro confirmation incubations using the selected potential candidates from each cycle of the screening test were performed to validate the results. In this assay, there were four replications of the control, monensin, and each candidate at a lower dosage of 10 mg. The fermentation run was repeated on three different days to check consistency.

### 2.3. Measurements and Chemical Analysis

After 24 h of incubation, the total gas volume in the headspace of the bottle was measured using a water displacement apparatus [27]. A gas sample was transferred to a 10-mL vacuum tube (ref 364979, BD Vacutainer, Becton Dickinson, Franklin Lakes, NJ, USA) for CH_4_ analysis. Then, the bottles were placed on ice to stop fermentation, the incubation medium was transferred to a 50-mL beaker, and the pH was measured using a pH meter (model AG 8603; Seven Easy pH, Mettler-Toledo, Schwerzenbach, Switzerland). For the microbial analysis, a 10-mL sample of incubation medium was stored at −80 °C until DNA was extracted. The remaining medium was centrifuged at 12,000× *g* for 10 min (Centrifuge Smart 15, Hanil Science Industrial, Seoul, Korea), and the supernatant was stored at −20 °C to determine the ammonia nitrogen (NH_3_-N) and volatile fatty acid (VFA) concentrations.

CH_4_ concentration in the headspace gas was determined using the Agilent 7890B GC system (Agilent Technologies, Santa Clara, CA, USA) with a flame ionization detector. The inlet and detector temperature were maintained at 200 °C and 250 °C, respectively. A 10-mL sample was injected through the back inlet using a 10-mL graduated syringe connected to a two-way stopcock (KOVAX, Seoul, Korea) with a split ratio of 10:1 into a 30 m × 0.53 mm × 20 μm HayeSep Q–ValcoPLOT fused-silica capillary column (CFS-PQ3053-200, VICI Metronics, Danvers, MA, USA). The carrier gas helium (99.99%; Air Korea) was set to a flow rate of 10 mL/min and the oven temperature of 80 °C was held constant for 2.5 min. CH_4_ content was calculated by external calibration, using a certified gas mixture (8% mol/mol balance N_2_; Air Korea). The NH_3_-N concentration was determined using a modified colorimetric method [28]. For VFA analysis, 5.0-mL aliquot of sample was mixed with 1.0 mL 25% HPO_3_ and 0.2 mL 2% pivalic acid [29], then analysed using gas chromatography as described previously to identify the VFAs [30]. The feed and substrate samples were dried in a forced-air oven at 65 °C for 72 h to estimate DM content and then ground to pass through a 1-mm screen (Model 4, Thomas Scientific, Swedesboro, NJ, USA). Nutrient compositions were determined using methods described previously [30].

### 2.4. Analysis of Plant Secondary Metabolites

Total phenols, total tannins, and condensed tannins were determined in the selected crude extracts based on the method described by Makkar [31]. For extraction, 60 mg of crude methanol or ethanol extract was mixed with 3.5 mL of aqueous acetone (70:30 *v/v*), vortexed, and incubated at room temperature for 1 h. Subsequently, the mixture was centrifuged at 3000× *g* (Hanil Science Industrial, Gimpo, Korea) for 10 min, and the supernatant was collected and used for assays. Total phenols and total tannins were expressed as catechin (CAS No. 225937-10-0, Sigma-Aldrich) equivalents and condensed tannins were expressed as cyanidin (CAS No.528-58-5, Sigma-Aldrich) equivalents. Total tannic acids or hydrolysable tannins (HTs) were estimated as the difference between total tannins and condensed tannins [32]. Total saponin (TS) content was determined [33], and expressed as escin (CAS No. 6805-41-0, Sigma-Aldrich) equivalents. PSMs were expressed as units per milligram of extract, because the DM contents of the plant parts and extraction yield were unknown.

### 2.5. Gas Chromatography-Mass Spectrometry (GC-MS) Analysis

Seeds of *Pharbitis nil* (100 g) were ground and extracted with 1000 mL of ethanol (98%) for 24 h at room temperature in an orbital shaker. The extract was filtered through Whatman No. 2 filter paper and concentrated using a rotary vacuum evaporator (Heidolph Instruments, Schawabatch, Germany). The resulting extract (without derivatization) was diluted 10-fold, and the GC-MS analysis was performed using a TSQ 8000 triple quadrupole MS interfaced with a TRACE 1310 GC (Thermo Scientific, Waltham, MA, USA) equipped with a TG-5MS (30 × 0.25 mm× 0.25 μm; Agilent Technologies) 5%-phenyl-methylpolysiloxane fused capillary column. Pure helium gas (99.99%; Air Korea) was used as the carrier gas at a constant flow rate of 1.2 mL/min and a splitless injection volume of 1 μL. The injector temperature was maintained at 280 °C and oven temperature was programmed from 80 °C (isothermal for 2 min), with an increase of 15 °C/min to 250 °C (isothermal for 5 min), then 15 °C/min to 300 °C, ending with a 4-min isothermal incubation at 300 °C. Mass spectra were collected at 70 eV with a scan-interval of 1.0 s and fragments ranging from 50 to 550 *m/z*. The solvent delay was 0 to 2 min, and total run time was 25 min. Phytochemicals present in the extracts were identified based on a comparison of their mass spectral patterns with the spectral database at the library of the National Institute of Standards and Technology (NIST, Gaithersburg, MD, USA).

### 2.6. DNA Extraction and Real-Time PCR

Genomic DNA from the incubation medium was extracted using the NucleoSpin soil kit (Macherey-Nagel, DuÈren, Germany), and nucleic acid concentrations were measured as described previously [30]. The integrity of the gDNA was confirmed by visualising the bands using eco dye-stained (Biofact, Seoul, Korea) agarose gel electrophoresis. Real-time PCR assays to determine the relative abundances of major cellulolytic bacteria, such as *Ruminococcus albus, Ruminococcus flavefaciens, Fibrobacter succinogens*, total methanogens, and ciliated protozoa were performed using the SYBR Green real-time-PCR Master Mix (Bioneer, Daejeon, Korea) and the CFX96 Touch™ Real-Time PCR Detection System (Bio-Rad Laboratories Inc., Hercules, CA, USA). Thermal cycling was performed based on the annealing temperature that showed high product band intensity and determined by multiple gradient PCR for each primer set as shown in Table 3. The primers targeted the 16 s or 18 s variable region for relative quantification. Briefly, the PCR was carried out in 20-μL total reaction volumes containing 20 ng gDNA, 10 μL SYBR Green RT-PCR Master Mix, and 1.0 μL of each 10-μM primer. Thermal cycling consisted of initial denaturation at 95 °C for 10 min, followed by 40 cycles at 94 °C for 15 s and annealing for 30 s followed by extension at 72 °C for 30 s [34]. The annealing was carried out at specific temperatures corresponded for each primer sets as mentioned in Table 3. After an amplification cycle, a melting curve analysis was performed starting at 65 °C with an increase of 0.5 °C to 95 °C, followed by a plate read. The 2^−ΔΔCT^ method was used to determine the relative fold-changes [35], and all data were normalised to the abundance of total bacteria.

### 2.7. Statistical Analysis

In screening assay, Student’s *t*-test was used to compare the total gas and CH_4_ production levels in the control bottles with those levels in bottles containing a given plant additive from the same incubation run. The effects were expressed as relative change to the value of the control for the specific incubation run. The confirmation assay results were analysed using one-way analysis of variance, followed by Newman–Keuls multiple comparison tests. All statistical analyses were performed using GraphPad Prism, version 5.0 (GraphPad Software Inc., La Jolla, CA, USA), and a *p*-value < 0.05 was considered statistically significant. To identify bacterial lineages and other parameters that differentiated the control and treatment groups, we performed principal component analysis using the fviz_pca_biplot function in the FactoMineR [39] package of R-software, version 4.0.3 (The R Foundation for Statistical Computing, Vienna, Austria). The non-parametric Kendall rank-correlation coefficient was calculated to identify correlations among CH_4_ production, fermentation characteristics, bacterial communities, and PSMs using the PROC CORR function in SAS software, version 9.4 (SAS Institute, Cary, NC, USA).

## 3. Results and Discussion

While many strategies have been proposed to mitigate enteric CH_4_ [2,3], most (e.g., defaunation, direct-fed microbials, ionophores, and bacteriocins) are difficult to implement at the farm level due to practical difficulties. Therefore, dietary manipulations, such as plant-based anti-methanogenic feed additives, offer highly effective CH_4_ mitigation approaches [4,13,14,15,16,19,40,41,42]. In vitro experimental models are very useful for the preliminary screening of a large number of plant additives to select a few potent additives with desired characteristics. Plants are either directly used in the reaction mixture [20,21,22] or used as dry extracts during the screening process [23]. Therefore, we initially obtained 152 dry methanolic or ethanolic extracts of 137 plant species that are widely distributed in Korea and could be readily available as potential feed additives.

**Figure 1:**
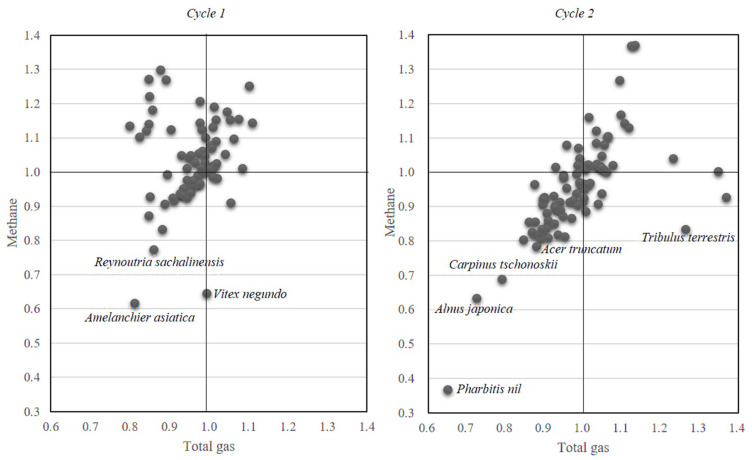
Relative changes in total gas production and CH_4_ concentration in gas, respective to their controls, after 24-h in vitro incubation with plant extracts (replicate = 3). Control is considered as 1.

The relative effects of each plant extract on total gas and CH_4_ production (mmol per g of DM) during a screening assay conducted during the two different cycles are shown in Figure 1. CH_4_ production decreased by more than 10% in 20% of the extracts tested. Although the extracts from stems of *Acer tegmentosum* Maxim., leaves of *Carpinus laxiflora* (Siebold & Zucc.) Blume, leaves of *Cleyera japonica* Thunb., aerial parts of *Erigeron annuus* Pers., stems of *Taxus cuspidate* Siebold & Zucc., and stems of *Ginkgo biloba* L. exhibited a reduction of CH_4_ close to 20%, they were not included as candidates for the confirmation assay. Only eight extracts (5% of the extracts tested) reduced (*p* < 0.1) CH_4_ production by more than 20% (Supplementary Table S1) and were considered promising candidates for subsequent confirmation assays. These included stems of *Vitex negundo* var. *incisa* (Lam.) C.B. Clarke (VI), stems of *Amelanchier asiatica* (Siebold & Zucc.) Endl. ex Walp. (AM), fruit of *Reynoutria sachalinensis* Nakai (RE) from cycle 1, seeds of *Tribulus terrestris* L. (TR), seeds of *Pharbitis nil* (L.) Choisy (PA), leaves of *Alnus japonica* Siebold & Zucc. (AL), stems and bark of *Carpinus tschonoskii* Maxim. (CA), and stems of *Acer truncatum* Bunge (AC) from cycle 2. Among these, PA exhibited the maximum reduction of CH_4_ by 63%, compared to the control. Most potential plant extracts decreased (*p* < 0.1) total gas production by 12–35%, except VI and TR, which had a negligible effect (Supplementary Table S1). These results could be attributed to the dosage (20 mg) of the plant extracts, which may have had a detrimental effect on ruminal microbes. Thus, the plant extracts were tested at a relatively lower dosage (10 mg) in subsequent confirmation assays, compared to the dosage in screening assays.

The effects of the selected candidates on CH_4_, gas production, fermentation characteristics, and microbial abundance were confirmed in an in vitro assay (Table 4 and Table 5). Significant decreases (*p* < 0.05) in CH_4_ production (mmol per g of DM incubated) in response to adding VI (17%), AM (17%), RE (19%), TR (22%), PA (37%), AL (27%), AC (23%), and CA (23%) were observed at half extract concentrations, compared to the screening assay. This also corresponded to reductions of CH_4_ concentration in total gas of 7%, 11%, 9%, 9%, 15%, 11%, 10%, and 10% (*p* < 0.05), respectively, compared to the control.

**Figure 2:**
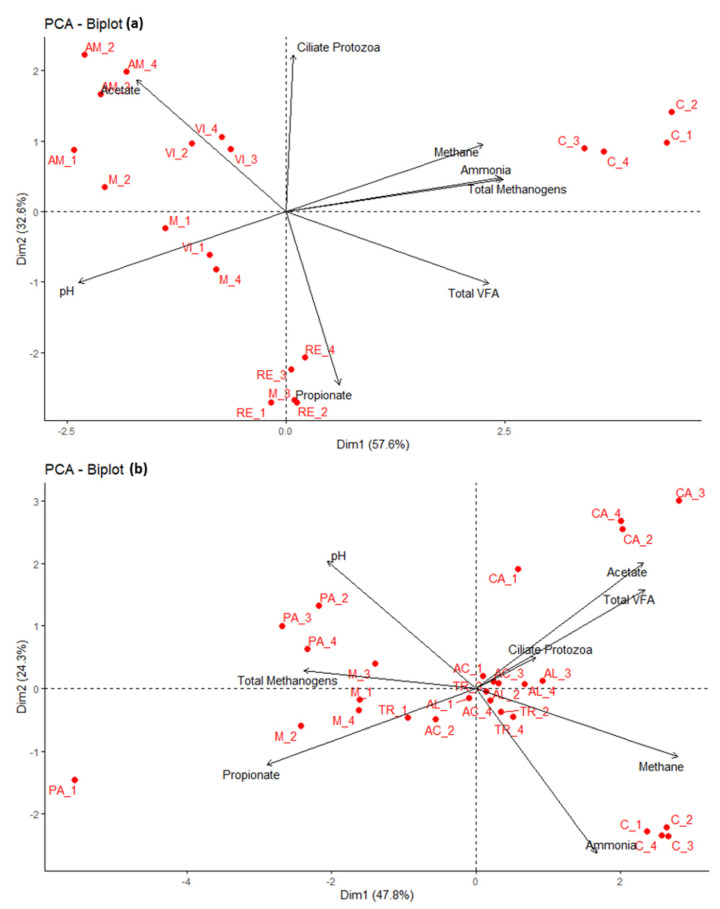
Principal components analysis of CH_4_ production, microbial diversity, and fermentation parameters in control and tested plant extracts during cycle 1 (**a**) and cycle 2 (**b**). Percentages of variation explained by PC1 and PC2 are indicated on the respective axes. C- control; M-monensin; VI-*Vitex negundo*; AM-*Amelanchier asiatica*; RE-*Reynoutria sachalinensis*; TR-*Tribulus terrestris*; AL-*Alnus japonica*; PA-*Pharbitis nil*; AC-*Acer truncatum*; CA-*Carpinus tschonoskii*.

Principal component analysis also discriminated the treatments from their respective controls, explaining 57.6% and 47.8% of variation during cycles 1 and 2, respectively (Figure 2). Furthermore, this is the first study to report the anti-methanogenic activities of these extracts, although reports regarding such activities are available for leaves of VI [43], and gross saponins from TR [44]. However, the extents of CH_4_ mitigation in previous studies might not be comparable with the extent in the current study because of the different plant parts and dosages used. In addition, despite the lower dose of supplemented extracts compared to the screening assay, an increase (*p* < 0.05) in pH and decreases (*p* < 0.05) in total gas production, total VFA, and NH_3_-N were detected in the confirmation assay. A higher pH and reduced VFA concentrations are indications of overall inhibition of rumen microbial fermentation, which would not be nutritionally beneficial to the host animal, since VFAs are major energy source for the ruminants [45]. However, this effect is comparable with the effect of monensin, suggesting that the extracts have similar properties to those of monensin. This could be attributed to the greater concentrations of PSMs in the tested extracts, which are known for their anti-microbial activities [46]. Most of the plant extracts tested in this study (except seeds of TR and PA) were rich in total phenols, total tannins, HTs, and TSs (Table 6). This is consistent with previous studies reporting greater concentrations of polyphenols, flavonoids, and saponins in tested plant species with anti-microbial properties [47,48,49,50,51,52]. It has also been reported that HTs reduce the production of total VFAs through actions on ruminal microbes [53,54]. This is further supported by the significant decrease (*p* < 0.001) in the *F. succinogens* population in this experiment (Table 5), which is an efficient producer of succinate and the major precursor for propionate synthesis [55].

Similarly, the decrease in NH_3_-N might be related to proteolysis inhibition through the formation of insoluble tannin–protein complexes [56,57]. Getachew et al. [58] reported a decrease in protein degradation and NH_3_-N after supplementation with tannic acids. This finding suggests that the addition of a tannin-rich extract might minimise the degradability of protein in the rumen and exert beneficial effects similar to those that occur when ruminants are supplemented with rumen undegradable protein (reviewed in [59]). Hydrolysable tannins with low molecular weight and less structural variability than condensed tannins result in more consistent reduction of CH_4_ due to gallic acid subunit binding to methanogens [60]. In the current study, the HT concentration provided by the extracts (1.15–1.35 g/100 g DM) was comparable with the level (1.43 g/100 g DM) supplemented in the study by Aboagye et al. [60], who observed a 9% decrease in CH_4_ yield. In addition, Jayanegara et al. [61] showed that HTs decrease the methanogen population and microbes, which provide H_2_ to a greater extent, compared to condensed tannins. Pure saponins and saponin-containing plants or extracts have inhibitory effects on protozoans (reviewed in [62]), which contribute to CH_4_ production via interspecific H_2_ transfer to methanogens [63]. In the current study, the abundances of total methanogens in VI, AM, and RE decreased (*p* < 0.001), as did ciliated protozoa in VI and RE (*p* < 0.001), compared to the control (Table 4). These findings clearly showed the effects of HTs and TSs on H_2_ and CH_4_ production, which thereby affect total gas production. These findings were supported by stronger negative (τ = −0.51, *p* = 0.070) and positive (τ = 0.64, *p* < 0.05) correlations between TS content and CH_4_ production, and protozoan abundance and gas production, respectively (Table 7). However, AL, AC, and CA reduced CH_4_ without any negative effects on methanogens or the protozoan population, compared to the control (Table 5). Expression analysis of methyl-co reductase (*MCR*) gene can provide a better understanding of complex methanogenesis processes than methanogen abundance analyses based on 16s rDNA [64]. Other studies have also demonstrated that CH_4_ production is not correlated with methanogens abundance, but with its composition (reviewed in [65]). Furthermore, saponins may decrease the activities of CH_4_ producing genes or the rate of CH_4_ production in methanogenic cells [66], suggesting that PSMs from different sources have different effects on microbes and methanogenesis [67]. However, directly or indirectly inhibiting CH_4_ production entails a change in the VFA profile, mostly favouring greater propionate production [68]. Gram-positive ruminal bacteria generally produce acetate and butyrate, while Gram-negative bacteria produce propionate [69]. The decrease in CH_4_ production caused by most of the tested extracts in this experiment, without any changes in the proportions of individual VFAs (except PA), suggests broad spectral antibacterial activities of PSMs targeting Gram-positive and negative bacteria. However, no negative effects were observed on selected microbes, such as *R. flavefaciens* and *R. albus*, in this experiment. Some studies have reported that PSMs target other ruminal microbes with minimal effects on *Ruminococcus* spp. (reviewed in [46]).

Despite the ban on the non-therapeutic use of monensin in the Republic of Korea, it remains one of the most commonly used ionophores in ruminants in other countries. Monensin supplementation has been associated with decreased methanogenesis accompanied by improved feed digestibility, increased propionate synthesis, and decreased NH_3_-N production [70]. A recent study [71] also showed a decrease in CH_4_ production coupled with a decrease in H_2_-producing microorganisms (e.g., protozoa, fungi, and Gram-positive *Firmicutes*) after supplementation with monensin. Intriguingly, in the current study, the decrease in CH_4_ production caused by PA alone was accompanied by decreases in protozoan abundance and NH_3_-N concentration, as well as an increase in the proportion of propionate, similar to the effect of monensin (Table 5).

Principal component analysis grouped PA and monensin, explaining 47.8% of the variation from their respective controls (Figure 2b). The PCA analysis also exhibited a strong correlation of propionate towards PA and monensin, further supporting our statement. The observed effect of PA with a very low concentration of TSs and near absence of HTs suggests the presence of other potentially bioactive compounds in PA. GC-MS analysis revealed the presence of a heterogeneous mixture, dominated by polyunsaturated fatty acids (Table 8). Seeds of *P. nil* had greater concentrations of 9,12-octadecadienoic acid (Z,Z)- (23%), commonly known as linoleic acid (18:2), followed by 9,12-octadecadienoic acid (Z,Z)-,2,3-dihydroxypropyl ester (18%) commonly known as alpha-glyceryl linoleate. Overall, 60% of the compounds identified were classified either as fatty acids or fatty acid amides. A meta-analysis by Patra et al. [19] established negative associations between total dietary polyunsaturated fatty acid concentrations and CH_4_, VFAs, and NH_3_-N production in the rumen. The effects of polyunsaturated fatty acids on CH_4_ production were attributed to the change in H_2_ thermodynamics in the rumen, caused by inhibition of protozoa, biohydrogenation of unsaturated fatty acids, and increased production of propionic acid, which compete with methanogenesis for metabolic H_2_ [72,73]. A strong negative association (τ = −0.51, *p* = 0.070) was noted between protozoan abundance and propionate proportion in the current study. A meta-analysis by Guyader [74] reported a decrease in protozoan abundances in experiments supplemented lipids on ruminants’ diet, which was due to changes in membrane permeability, resulting in cell lysis [75]. In addition, Dohme et al. [76] reported a detrimental effect of linoleic acid (18:2) on the protozoan and total bacterial populations. This is consistent with the decreased (*p* < 0.001) abundance of the ciliated protozoa, *R. flavefaciens* and *F. succinogens* in PA, in the current study. However, complete metabolite profile of PA using chromatographic techniques with proper derivatization procedures would give deeper understanding of the compound responsible for the action. Moreover, enrichment of dietary linoleic acid (18:2), a precursor of bioactive conjugated linoleic acids [77], suggested that PA seeds might be a promising feed additive for ruminants. In addition, PA seeds have been widely used in Korean and Chinese traditional medicine for their roles in improving digestibility and intestinal motility (reviewed in [78]). Therefore, PA seeds could act as a source of fatty acids, probably replacing oil seeds that have been reported to decrease DM and neutral detergent fibre digestibility [19]. However, future in vitro or in vivo trials are needed to confirm their effects on rumen nutrient digestibility and animal performance, since the protozoal defaunation was associated with decrease in rumen organic matter digestibility and specifically NDF and ADF digestibility [79].

## 4. Conclusions

The extracts rich in phenolic compounds from stems of *A. asiatica*, fruit of *R. sachalinensis*, seeds of *T. terrestris*, leaves of *A. japonica*, stems and bark of *C. tschonoskii*, and stems of *A. truncatum* reduced CH_4_ production and fermentation rates in vitro. The negative effects on total gas and VFA production suggest the need to standardise the doses of plant extracts that are effective for inhibiting CH_4_ emissions with minimum adverse effects on fermentation. These supplemental plant extracts seem to decrease the output of ammonia from protein degradation, although the post ruminal nitrogen use efficiency is still remained to be elucidated in ruminants. Notably, the maximum reduction in CH_4_ production by the extracts from the seeds of *P. nil*, which are rich in linoleic acid (18:2) and other fatty acid amides, is a promising alternative to ionophores and oilseeds to mitigate CH_4_ emissions. In vivo trials must be conducted to elucidate the adaptation of rumen microbes to the seeds of *P. nil* over a prolonged feeding period.

## Figures and Tables

**Table 1:** Scientific names, common names, and parts of plants screened in the in vitro assay.

Scientific Names	Common Names	Parts Used ^1^
*Abelia mosanensis T.H.Chung ex Nakai*	Sweet abelia	Stem
*Abeliophyllum distichum* Nakai	White forsythia	Stem
*Abies koreana* E.H.Wilson	Korean fir	Leaf
*Abies koreana* E.H.Wilson	Korean fir	Stem
*Acanthopanax senticosus* (Rupr. & Maxim.) Harms	Siberian ginseng	Leaf, stem
*Acer palmatum* Thunb.	Japanese maple	Leaf
*Acer pictum* subsp. *mono*(Maxim.) H. Ohashi	Painted maple	Leaf
*Acer pseudo-sieboldianum* var. *koreanum* Nakai	Korean maple	Leaf, stem
*Acer takesimense* Nakai	Takeshima Korean maple	Leaf, stem
*Acer tataricum* subsp. *ginnala* (Maxim.) Wesmael	Tatarian maple	Stem
*Acer tegmentosum* Maxim.	Manchurian striped bark maple	Stem
*Acer triflorum* Kom.	Three-flowered maple	Stem
*Acer truncatum* Bunge	Shangtung maple	Stem
*Acer tschonoskii* var. *rubripes* Kom.	Butterfly maple	Stem
*Aconitum carmichaeli* Debeaux	Carmichael’s monkshood	Tuber [E]
*Actinodaphne lancifolia* (Sieb. & Zucc.) Meisn	Unknown	Leaf
*Adonis amurensis* Regel & Radde	Amur adonis	Aerial parts
*Allium grayi* Regel	Long-stamen chive	Aerial parts
*Alnus japonica* Siebold & Zucc.	Japanese alder	Leaf
*Alnus maximowiczii* Callier ex C.K.Schneid.	Montane alder	Leaf
*Amelanchier asiatica* (Sieb. & Zucc.) Endl. ex Walp.	Korean juneberry	Stem
*Amomum villosum* Lour.	Bastard cardamon	Fruit [E]
*Ampelopsis japonica* (Thunb.) Makino	Peppervine	Tuber [E]
*Angelica japonica* A.Gray	Unknown	Leaf
*Angelica japonica* A.Gray	Unknown	Stem, root
*Aralia continentalis* Kitag.	Manchurian spikenard	Stem
*Ardisia crenata* Sims.	Coral ardisia	Leaf
*Ardisia japonica* (Thunb.) Blume	Marlberry	Leaf
*Ardisia japonica* (Thunb.) Blume	Marlberry	Stem
*Areca catechu* L.	Betelnut palm	Pericarp [E]
*Arisaema takesimense* Nakai	Cobra lily	Stem
*Asarum sieboldii* Miq.	Wild ginger	Aerial parts
*Atractylodes macrocephala* Koidz.	Bai Zhu	Rhizome [E]
*Aucuba japonica* Thunb.	Spotted laurel	Leaf
*Callicarpa japonica* var. *leucocarpa* Siebold	Japanese beautyberry	Fruit
*Calystegia soldanella*(L.) R.Br.	Sea bindweed	Aerial parts
*Camellia japonica* L.	Japanese camellia	Stem
*Camellia japonica* L.	Japanese camellia	Leaf
*Campanula takesimana* Nakai	Korean bellflower	Aerial parts
*Capsella bursa-pastoris*(L.) Medik.	Shepherd’s purse	Aerial parts
*Cardamine amaraeformis* Nakai	Brewer’s bittercress	Aerial parts
*Cardamine flexuosa* Withering	Wavy bittercress	Stem
*Carpinus laxiflora* (Siebold & Zucc.) Blume	Hornbeam	Leaf
*Carpinus laxiflora* (Siebold & Zucc.) Blume	Hornbeam	Stem, bark
*Carpinus tschonoskii* Maxim.	Silky hornbeam	Stem, bark
*Castanopsis cuspidata* var. *sieboldii* (Makino) Nakai	Japanese chinquapin	Stem, heart wood
*Celtis choseniana* Nakai	Hackberry	Stem
*Cephalotaxus koreana* Nakai	Korean plum yew	Leaf
*Chaenomeles lagenaria* (Loisel.) Koidz.	Flowering quince	Stem
*Cinnamomum camphora*(L.) J.Presl	Camphor laurel	Leaf
*Citrus dachibana* (Makino) Tanaka.	Tachibana orange	Stem, bark
*Cleyera japonica* Thunb.	Sakaki	Leaf
*Cornus controversa* Hemsl.	Giant dogwood	Stem
*Corydalis incisa* Pers.	Fumewort	Aerial parts
*Corylus heterophylla* var. *thunbergii* Blume	Siberian filbert	Leaf, stem
*Crataegus pinnatifida* Bunge	Mountain hawthorn	Stem
*Daphne genkwa* Siebold & Zucc.	Lilac Daphne	Stem, root
*Dioscorea tokoro* Makino	Unknown	Rhizome [E]
*Dolichos lablab* L.	Hyacinth bean	Seed [E]
*Elaeagnus glabra* Thunb.	Goat nipple	Stem
*Elaeagnus umbellate* C.P.Thunb. ex A.Murray	Autumn olive	Leaf, stem
*Equisetum arvense* L.	Horsetail	Aerial parts [E]
*Erigeron annuus* (L.) Pers.	Annual fleabane	Aerial parts
*Eriobotrya japonica* (Thunb.) Lindl.	Japanese medlar	Leaf
*Euphorbia helioscopia* L.	Sun spurge	Aerial parts
*Euphorbia sieboldiana* C.Morren & Decne.	Unknown	Aerial parts
*Eurya emarginata* (Thunb.) Makino	Shore eurya	Leaf
*Ficus erecta* Thunb.	Japanese fig	Fruit
*Ficus nipponica* Franch. & Sav.	Japanese fig	Stem
*Forsythia nakaii*(Uyeki) T.B.Lee	*Unknown*	Stem
*Ginkgo biloba* L.	Common gingko	Stem
*Hedera rhombea*(Miq.) Siebold ex Bean	Japanese ivy	Leaf
*Hedera rhombea*(Miq.) Siebold ex Bean	Japanese ivy	Fruit
*Hedera rhombea*(Miq.) Siebold ex Bean	Japanese ivy	Aerial parts
*Hedera rhombea*(Miq.) Siebold ex Bean	Japanese ivy	Stem
*Hepatica insularis* Nakai	Unknown	Aerial parts
*Heracleum moellendorffii* f. *Subbipinnatum* (Franch.) Kitag.	Cow parsnip	Leaf
*Hydrangea serrata* f. *acuminate* (Siebold & Zucc.) E.H.Wilson	Mountain hydrangea	Stem
*Hydrangea serrata* f. *acuminate* (Siebold & Zucc.) E.H.Wilson	Mountain hydrangea	Leaf, stem
*Ilex cornuta* Lindl. & Paxton	Chinese holly	Leaf
*Ilex crenata* var. *microphylla* Maxim.	Japanese holly	Stem
*Illicium religiosum* Siebold & Zucc.	Japanese star anise	Stem
*Juniperus rigida* Pav. ex Carrière	Needle juniper	Leaf
*Juniperus rigida* Pav. ex Carrière	Needle juniper	Stem
*Kirengeshoma koreana* Nakai	Yellow waxbells	Stem
*Kirengeshoma koreana* Nakai	Yellow waxbells	Root
*Koelreuteria paniculata* Laxm.	Golden raintree	Stem
*Lathyrus japonicas* Willd.	Beach pea	Aerial parts
*Ligularia fischeri* (Ledeb.) Turcz.	Fischers ragwort	Aerial parts
*Lindera erythrocarpa* Makino	Asian spicebush	Stem
*Lindera obtusiloba* Blume	Japanese spicebush	Leaf, stem
*Litsea japonica* Mirb.	Unknown	Leaf
*Lonicera japonica* Thunb.	Chinese honeysuckle	Leaf
*Lonicera japonica* Thunb.	Chinese honeysuckle	Stem
*Lonicera vesicaria* Kom.	Korean honeysuckle	Leaf, stem
*Lotus corniculatus* var. *japonicus* Regel	Bird’s foot trefoil	Aerial parts
*Luzula capitate* (Miq. ex Franch. & Sav.) Kom.	Sweep’s woodbrush	Aerial parts
*Lycoris squamigera* Maxim.	Magic-lily	Leaf
*Lycoris squamigera* Maxim.	Magic-lily	Stem
*Machilus japonica* Siebold & Zucc.	Unknown	Twig
*Meehania urticifolia* (Miq.) Makino	Japanese dead nettle	Aerial parts
*Megaleranthis saniculifolia* Ohwi	Unknown	Aerial parts
*Melia azedarach* var. *japonica* (G.Don) Mak.	Bead tree	Aerial parts
*Morus bombycis* Koidz.	Korean mulberry	Leaf
*Orostachys iwarenge* (Makino) Hara	Chinese Dunce cap	Aerial parts
*Osmanthus insularis* Koidz.	Holly olive	Leaf
*Pharbitis nil* (L.) Choisy	Japanese morning glory	Seed [E]
*Pinus parviflora* Siebold & Zucc.	Japanese white pine	Leaf
*Pinus thunbergii* Parl.	Japanese black pine	Leaf
*Pittosporum tobira* (Murray) Aiton fil.	Japanese mock orange	Stem
*Potentilla fruticosa* L.	Shrubby cinquefoil	Stem
*Pourthiaea villosa* (Thunb.) Decne.	Oriental Photinia	Stem
*Prunus sargentii* Rehder	Sargent’s cherry	Stem
*Pyrus calleryana* var. *fauriei* (C.K.Schneid.) Rehder	Fauriei callery pear	Stem
*Quercus acuta* Siebold ex Blume	Japanese evergreen oak	Stem
*Quercus aliena* Blume	Oriental white oak	Leaf, stem
*Quercus gilva* Blume	Redbark oak	Leaf
*Quercus gilva* Blume	Redbark oak	Stem, heart wood
*Reynoutria sachalinensis* (F.Schmidt) Nakai	Sakhalin knotweed	Fruit
*Rhodotypos scandens* (Thunb.) Makino	Black jetbead	Stem
*Rhus trichocarpa* Miq.	Bristly-fruit lacquer tree	Stem
*Rosa multiflora* Murray	Many-flowered Rose	Leaf, stem
*Salix glandulosa* Seemen	Korean king Willow	Stem
*Salix hulteni* Flod.	Hulten Willow	Stem
*Sambucus sieboldiana* var. *pendula* (Nakai) T.B.Lee	Japanese red elder	Stem
*Saussurea lappa*(Decne.) C.B.Clarke, 1876	Indian costus	Root [E]
*Sinapis alba* L.	White mustard	Seed [E]
*Sorbus alnifolia* (Sieb. & Zucc.) C.Koch	Korean mountain ash	Stem
*Spiraea salicifolia* L.	Bridewort	Stem
*Spirodela polyrhiza* (L.) Schleid.	Common duckmeat	Aerial parts [E]
*Staphylea bumalda* DC.	Bumalda bladdernut	Stem
*Strychnos nux-vomica* L.	Nux-vomica	Seed [E]
*Styrax obassia* Siebold & Zucc.	Fragrant snowbell	Stem
*Taxus cuspidate* Siebold & Zucc.	Japanese yew	Stem
*Thea sinensis* L.	Chinese tea	Leaf
*Torreya nucifera* Siebold & Zucc.	Japanese nutmeg tree	Stem
*Trachelospermum asiaticum* var. *intermedium* Nakai	Chinese jasmine	Leaf
*Trachelospermum jasminoides* (Lindl.) Lem.	Star jasmine	Stem, leaf [E]
*Tribulus terrestris* L.	Puncture vine	Leaf [E]
*Tribulus terrestris* L.	Puncture vine	Seed [E]
*Triticum aestivum* L.	Common wheat	Seed [E]
*Tsuga sieboldii* Carrière	Japanese hemlock	Leaf
*Vaccinium bracteatum* Thunb.	Sea bilberry	Leaf
*Viburnum awabuki* Hort.Berol. ex C.Koch	Sweet viburnum	Leaf
*Viburnum carlesii* Hemsl. ex Forb. & Hemsl.	Korean spice viburnum	Stem
*Viburnum sargentii* Koehne	Sargent viburnum	Stem
*Vicia angustifolia* var. *segetalis* (Thuill.) W.D.J.Koch	Black-pod vetch	Aerial parts
*Viola japonica* Langsd. ex DC.	Japanese violet	Aerial parts
*Viola tokubuchiana* var. *takedana* (Makino) Maek.	Unknown	Aerial parts
*Vitex negundo* var. *incisa* (Lam.) C.B.Clarke	Chinese chaste tree	Stem
*Vitis coignetiae* Pulliat ex Planch.	Crimson gloryvine	Stem
*Youngia denticulata* (Houtt.) Kitam.	Unknown	Aerial parts

^1^ Unless indicated otherwise, methanol (95%) was used for extraction. [E], ethanol (95%) used for extraction.

**Table 2:** Ingredients and chemical composition of substrate used in the in vitro screening and confirmation assays.

Ingredient Composition	g/kg DM
Timothy hay	46
Klein grass	31
Oat hay	31
Alfalfa hay	73
Tall fescue grass	69
Rye grass	38
Cotton seed	43
Beet pulp	77
Corn gluten feed	136
Dried brewers’ grains	195
Commercial concentrate	230
Vitamin-Mineral premix ^1^	23
Probiotics	9
**Chemical Composition**	**g/kg DM**
Organic matter	910
Crude protein	143
Ether extract	38
Neutral detergent fibre ^2^	289
Acid detergent fibre ^3^	143
Gross energy, MJ/kg DM	17.7

^1^ Provided following nutrients per kg of mixture (Grobic-DC, Bayer Health Care, Leverkusen, Germany): Vit. A, 2,650,000 IU; Vit. D3, 530,000 IU; Vit. E, 1050 IU; Niacin, 10,000 mg; Mn, 4400 mg; Zn, 4400 mg; Fe, 13,200 mg; Cu, 2200 mg; I, 440 mg; Co, 440 mg. ^2^ Neutral detergent fibre assayed with a heat stable amylase and expressed exclusive of residual ash. ^3^ Acid detergent fibre expressed excluding residual ash

**Table 3:** Oligonucleotide primers used for real-time PCR assay.

Target Group	Primer Sequence	T_m_ (°C)	Size (bp)	Reference
Total bacteria	F: CGG CAA CGA GCG CAA CCC	60.5	130	[36]
	R: CCA TTG TAG CAC GTG TGT AGC C			
*Fibrobacter succinogenes*	F: GTT CGG AAT TAC TGG GCG TAA A	51.7	120	[36]
	R: CGC CTG CCC CTG AAC TAT C			
*Ruminococcus albus*	F: CCC TAA AAG CAG TCT TAG TTC G	47.0	176	[37]
	R: CCT CCT TGC GGT TAG AAC A			
*Ruminococcus flavefaciens*	F: CGA ACG GAG ATA ATT TGA GTT TAC TTA GG	53.3	132	[36]
	R: CGG TCT CTG TAT GTT ATG AGG TAT TAC C			
Total methanogens	F: CCGGAGATGGAACCTGAGAC	52.6	165	[38]
	R: CGGTCTTGCCCAGCTCTTATTC			
Ciliate protozoa	F: GAG CTA ATA CAT GCT AAG GC	46.2	180	[34]
	R: CCC TCA CTA CAA TCG AGA TTT AAG G			

**Table 4:** Effects of selected plant extracts from cycle 1 on CH_4_ production, rumen fermentation parameters, and microbial abundance after 24-h in vitro incubation (replicate = 4)

Item	Control	Monensin	*Vitex negundo*	*Amelanchier asiatica*	*Reynoutria sachalinensis*	SEM	*p*-Value
pH	6.0 ^b^	6.4 ^a^	6.4 ^a^	6.4 ^a^	6.4^a^	0.04	<0.001
Gas, mmol/g DM substrate	11.2	9.3	10.01	10.3	9.9	0.49	0.158
CH_4_, mmol/g DM substrate	1.5 ^a^	1.1 ^b^	1.3 ^b^	1.2 ^b^	1.2 ^b^	0.07	0.018
CH_4,_ mmol/mol gas	134.1 ^a^	121.3 ^b^	124.6 ^b^	119.9 ^b^	121.9 ^b^	1.94	0.001
Total VFAs, mM	166.0 ^a^	126.8 ^b^	127.6 ^b^	127.1 ^b^	127.7 ^b^	6.83	0.003
Acetate (C_2_), %	57.3	57.5	58.4	58.3	58.4	1.74	0.987
Propionate (C_3_), %	24.8	25.8	25.0	25.1	25.0	1.10	0.999
Isobutyrate, %	1.0	1.0	1.0	1.0	1.0	0.03	0.980
Butyrate, %	12.2	11.1	11.2	11.3	11.2	1.20	0.965
Isovalerate, %	2.8	2.8	2.6	2.6	2.6	0.27	0.967
Valerate, %	2.0	1.9	1.9	1.8	1.8	0.23	0.981
C_2_/C_3_	2.3	2.2	2.3	2.3	2.3	0.09	0.933
NH_3_-N, mg/dL	28.5 ^a^	20.9 ^b^	19.9 ^b^	19.7 ^b^	19.4 ^b^	2.00	0.027
*Expression fold change*							
*R. flavefaciens*	1.0 ^d^	6.8 ^a^	3.6 ^c^	2.7^c^	5.2 ^b^	0.53	<0.001
*R. albus*	1.0 ^d^	5.4 ^c^	2.4 ^d^	6.0 ^cb^	12.7 ^a^	1.17	<0.001
*F. succinogenes*	1.0 ^c^	0.9 ^cd^	2.4 ^a^	2.3 ^ab^	1.0 ^cd^	0.21	<0.001
Total methanogens	1.0 ^a^	0.4 ^b^	0.3 ^c^	0.1 ^d^	0.3 ^c^	0.02	<0.001
Ciliate protozoa	1.0 ^b^	0.3 ^d^	0.7 ^c^	1.3 ^a^	0.3 ^d^	0.13	<0.001

Means with different superscripts differ significantly *p* < 0.05.

**Table 5:** Effects of selected plant extracts from cycle 2 on CH_4_ production, rumen fermentation parameters, and microbial abundance after 24-h in vitro incubation (replicate = 4)

Item	Control	Monensin	*Tribulus terrestris*	*Pharbitis nil*	*Alnus japonica*	*Acer truncatum*	*Carpinus tschonoskii*	SEM	*p*-Value
pH	6.1 ^b^	6.5 ^a^	6.4 ^b^	6.4 ^b^	6.4 ^b^	6.4 ^b^	6.4 ^b^	0.05	0.001
Gas, mmol/g DM substrate	12.7 ^a^	10.4 ^b^	10.6 ^b^	9.4 ^b^	10.5 ^b^	10.8 ^b^	10.6 ^b^	0.58	0.027
CH_4_, mmol/g DM substrate	1.8 ^a^	1.3 ^b^	1.4 ^b^	1.2 ^b^	1.4 ^b^	1.4 ^b^	1.4 ^b^	0.13	0.038
CH_4_, mmol/mol gas	144.8 ^a^	127.3 ^b^	131.6 ^b^	122.9 ^b^	128.7 ^b^	130.7 ^b^	130.6 ^b^	2.93	0.037
Total VFAs, mM	175.2 ^a^	132.2 ^b^	134.5 ^b^	133.1 ^b^	132.2 ^b^	130.7 ^b^	133.7 ^b^	9.84	0.044
Acetate (C_2_), %	55.3	54.4	56.3	53.0	56.4	56.4	56.5	3.47	0.988
Propionate (C_3_), %	21.1 ^a^	23.3 ^b^	21.8 ^ab^	27.1 ^b^	21.9 ^ab^	21.8 ^ab^	21.8 ^ab^	1.26	0.047
Isobutyrate, %	1.4	1.4	1.3	1.1	1.3	1.3	1.3	0.11	0.646
Butyrate, %	15.9	14.7	14.8	13.7	14.8	14.7	14.8	1.39	0.967
Isovalerate, %	3.6	3.7	3.4	3.2	3.3	3.3	3.3	0.31	0.913
Valerate, %	2.7 ^a^	2.6 ^abef^	2.5 ^abcef^	1.8 ^d^	2.4 ^e^	2.4 ^ef^	2.4 ^bcefg^	0.05	<0.001
C_2_/C_3_	2.6	2.4	2.6	2.0	2.6	2.6	2.6	0.17	0.097
NH_3_-N, mg/dL	42.9 ^a^	34.2 ^b^	33.4 ^b^	32.2 ^b^	30.8 ^b^	29.9 ^b^	28.4 ^b^	2.20	0.003
*Expression fold change*									
*R. flavefaciens*	1.0 ^g^	3.3 ^e^	4.9 ^d^	0.5^f^	6.25 ^c^	7.5 ^b^	10.2 ^ag^	0.76	<0.001
*R. albus*	1.0 ^g^	3.7 ^dfg^	4.7 ^bcdefg^	4.1 ^bcdefg^	13.02 ^a^	2.1 ^fg^	2.7 ^defg^	0.94	<0.001
*F. succinogenes*	1.0 ^a^	0.1 ^f^	0.3 ^e^	0.1 ^g^	0.50 ^cd^	0.7 ^b^	0.4 ^d^	0.06	<0.001
Total methanogens	1.0 ^e^	4.4 ^a^	0.9 ^efg^	3.4 ^b^	0.96 ^def^	1.3 ^def^	1.5 ^cd^	0.32	<0.001
Ciliate protozoa	1.0 ^c^	0.1 ^d^	16.9 ^a^	0.1 ^d^	12.2 ^b^	2.8 ^cd^	6.1 ^c^	1.56	<0.001

Means with different superscripts differ significantly *p* < 0.05.

**Table 6:** Contents of phenolic fractions and total saponins in extracts (mg/g crude extract; analytical replicate = 3).

Plant Species	Total Phenols	Non-Tannin Phenols	Total Tannins	Condensed Tannins	Hydrolysable Tannins	Total Saponins
*Vitex negundo*	93.8	2.7	91.1	10.0	81.1	216.0
*Amelanchier asiatica*	297.5	9.9	287.6	48.4	239.2	250.6
*Reynoutria sachalinensis*	213.0	4.6	208.4	19.3	189.0	243.3
*Tribulus terrestris*	11.9	0.4	11.5	*−*	11.5	115.8
*Pharbitis nil*	2.4	0.1	2.4	*−*	2.4	70.5
*Alnus japonica*	257.9	2.9	255.0	4.9	250.1	165.2
*Acer truncatum*	267.5	8.6	258.9	43.2	215.7	242.8
*Carpinus tschonoskii*	291.5	6.1	285.4	0.9	284.6	141.5

**Table 7:** Correlation coefficients between plant secondary metabolites, fermentation parameters, and microbial abundances (extract = 8).

	Ciliate Protozoa	Total Methanogens	*F. succinogenes*	*R. flavefaciens*	Total Saponins	Total Tannins	C_3_ (%)	C_2_ (%)	Total VFAs (mM)	CH_4_ (mmol/g DM)	Total Gas (mmol/g DM)
pH	0.43	0.40	−0.59 ^†^	0.28	−0.35	−0.04	−0.43	−0.67 *	0.59 ^†^	0.67 *	0.51 ^†^
Total gas (mmol/g DM)	0.64 *	0.18	−0.07	0.57 *	0.00	0.36	−0.71 *	−0.29	0.14	0.57 *	
CH_4_ (mmol/g DM)	0.50^†^	0.33	−0.36	0.43	−0.53 ^†^	−0.07	−0.57 *	−0.43	0.57 *		
Total VFAs (mM)	0.36	0.55†	−0.79 *	0.29	−0.71 *	−0.21	−0.43	−0.43			
C_2_ (%)	−0.36	−0.47	0.64 *	0.14	0.57 *	0.36	0.00				
C_3_ (%)	−0.51 ^†^	−0.40	0.21	−0.86 *	0.14	−0.36					
Total tannins	0.14	−0.11	0.29	0.50 ^†^	0.50 ^†^						
Total saponins	−0.21	−0.62 *	0.79	0.00							
*R. flavefaciens*	0.36	0.40	−0.07								
*F. succinogenes*	−0.29	−0.62 *									
Total methanogens	−0.04										

* *p* < 0.05; ^†^ *p* < 0.1.

**Table 8:** Bioactive compounds in ethanolic extracts from seeds of *Pharbitis nil* identified using GC-MS.

RT (min)	Compound	Formula	MW (g/mol)	Class	Area (%)
7.29	Ethanone, 1-(2-hydroxy-5-methylphenyl)-	C_9_H_10_O	150.2	Alkyl-phenylketone	6.2
8.49	(3-Nitrophenyl) methanol, n-propyl ether	C_10_H_13_NO_3_	195.2	Aromatic ether	11.9
12.23	Benzenepropanoic acid,3,5-bis(1,1-dimethylethyl)-4-hydroxy-, methyl ester	C_18_H_28_O_3_	292.4	Alkyl ester	6.6
12.31	l-(+)-Ascorbic acid 2,6-dihexadecanoate	C_38_H_68_O_8_	652.9	Fatty acid ester	6.1
13.44	9,12-Octadecadienoic acid (*Z,Z*)-	C_18_H_32_O_2_	280.4	PUFA ^1^	23.5
14.69	7,10-Hexadecadienoic acid, DMOX derivative	C_16_H_28_O_2_	252.4	LCFA ^2^	9.8
15.13	9-Octadecenamide, (*Z*)-	C_18_H_35_NO	281.5	Fatty amide	5.5
16.63	2,3-Dihydroxypropyl hexadecanoate	C_19_H_38_O_4_	330.5	Monoacylglycerol	6.7
19.21	9,12-Octadecadienoic acid (*Z,Z*)-,2,3-dihydroxypropyl ester	C_21_H_38_O_4_	356.5	Fatty amide	18.5
20.20	13-Docosenamide, (*Z*)-	C_22_H_43_NO	337.6	Fatty amide	2.3
24.21	ç-Sitosterol	C_29_H_50_O	414.0	Stigmastane	2.9

^1^ Polyunsaturated fatty acid; ^2^ Long chain fatty acid; MW molecular weight.

## Data Availability

All data generated in this study are included in the manuscript and Supplementary Files.

## Supplementary Materials

The following are available online at https://www.mdpi.com/article/10.3390/ani11041020/s1, Table S1: Relative changes in total gas production and methane concentration in gas in screening assay after 24 h of in vitro incubation (replicate = 3).
